# Assessing the Association of Hispanic Ethnicity and Other Personal Characteristics with Pharmacy School Admissions

**DOI:** 10.3390/pharmacy10040088

**Published:** 2022-07-20

**Authors:** Bernadette Cornelison, Christopher Edwards, David R. Axon, Lillian Gorman, Lorin Rudin-Rush, Bruce Johnson, Nancy A. Alvarez

**Affiliations:** 1 Department of Pharmacy Practice and Science, Tucson Campus, University of Arizona R. Ken Coit College of Pharmacy, Tucson, AZ 85721, USA; edwards@pharmacy.arizona.edu (C.E.); axon@pharmacy.arizona.edu (D.R.A.); johnson@pharmacy.arizona.edu (B.J.); 2 Department of Spanish and Portuguese, College of Humanities, University of Arizona, Tucson, AZ 85721, USA; lgorman@arizona.edu; 3 Department of Agricultural and Resource Economics, College of Agriculture and Life Sciences, University of Arizona, Tucson, AZ 85721, USA; lorinrr@arizona.edu; 4 Information Technology, University of Arizona R. Ken Coit College of Pharmacy, Tucson, AZ 85721, USA; 5 Department of Pharmacy Practice and Science, Phoenix Campus, University of Arizona R. Ken Coit College of Pharmacy, Phoenix, AZ 85004, USA; nalvarez@pharmacy.arizona.edu

**Keywords:** pharmacy, admissions, diversity

## Abstract

Access to healthcare services for underrepresented minority populations can be improved by having a diverse profession that reflects the diversity of the patient population being served. The criteria used for pharmacy school admissions may inhibit or strengthen the opportunities that URM students have to be accepted into the University of Arizona R. Ken Coit College of Pharmacy (COP) program. It is currently unclear how personal characteristics are associated with pharmacy school admissions at the COP. This study evaluates whether Hispanic ethnicity was associated with pharmacy school admission status, and secondarily, determines other characteristics associated with admissions. This retrospective database study used admissions data from 2005 to 2018. Completed applications were included in the analysis. The outcome variable was admitted versus not admitted to the pharmacy program. A multivariable logistic regression model was used to identify variables associated with admission status using an a priori alpha level of 0.05. A total of 2096 applicants were included in the analysis (mean age 25.1 ± 5.2 years, 59.9% female, 13.0% Hispanic). Hispanic ethnicity was not associated with admission status. Characteristics significantly associated with admission to pharmacy school were age, gender, high school attended, previous application to the college, and Pharmacy College Admission Test (PCAT) biology and chemistry scores. Although bias was not seen in the admissions process, this study highlights the need for intervention to ensure future cohorts better reflect the diversity of the region.

## 1. Introduction

Underrepresented minority (URM) populations in health professions in the U.S. include American Indians, Alaska Natives, Black or African Americans, Native Hawaiians or other Pacific Islanders races, as well as the non-homogenous Hispanic ethnic group [1]. In 2000, the U.S. Department of Health and Human Services reported that 3.2% of pharmacists practicing in the field of pharmacy were Hispanic [2]. In 2016, Hispanics made up the largest ethnic minority in the U.S.; however, Hispanic pharmacists made up only 4.5% of the 342,000 employed pharmacists across the country [3]. It has been well documented that having a diverse profession enhances communication and trust between patients and practitioners, can improve patient compliance with follow up visits, and promotes access to healthcare services for URM populations [4].

In 2019, Hispanics made up 31.7% of Arizona’s population [5]. In the city of Tucson, Arizona, home to the University of Arizona (UofA), Hispanics make up 43.2% of the population (548,073) and 28.3% of the undergraduate student population at UofA (36,503) [5,6]. The UofA is the state’s only land-grant university “whose mission is to improve the prospects and enrich the lives of the people of Arizona and the world through education, research, and creative expression” and sits on the original homeland of indigenous people who have stewarded the land for time immemorial [7]. In 2018, the UofA was designated a Hispanic Serving Institution (HSI) by the Department of Education. The UofA is the first 4-year public university in the state to receive this designation and one of four HSIs designated as research-intensive universities in the country [8]. Yet, this HSI designation does not define the notion of “servingness” and does not guarantee retention, student success or graduation of Hispanic students [9]. 

In 2020, the University of Arizona R. Ken Coit College of Pharmacy (COP) enrolled 536 Doctor of Pharmacy (PharmD) students. Of those, 111 students identified as Hispanic or Latino (20%) [6]. Students enrolled at the COP do not reflect the state’s nor city’s demographics and do not demonstrate a pipeline of success stemming from the university’s HSI designation. This study aims to provide insight into this observation.

The primary objective of this study was to determine whether Hispanic ethnicity was associated with pharmacy school admission status. The secondary objective was to identify any other variables associated with pharmacy school admission. 

## 2. Materials and Methods

This study utilized a retrospective database design. The COP used an online web-based application system during the time studied (2005–2018). In 2019, the admissions process was switched over to the Pharmacy College Application Service (PharmCAS) system and the admissions process was changed in 2020 and 2021 to accommodate the impact from the COVID19 pandemic. To avoid confounding results, data from 2019 to 2021 were excluded from the study. All data were input by applicants or college personnel during the application process. These data were saved during the admissions process in a set of relational database tables; data were extracted via anonymized queries. Subjects in the dataset were included if they had complete data available for all variables. Incomplete applications were excluded from the analysis.

The key independent variable was Hispanic status (Hispanic versus not Hispanic). All independent variables available from the database were included in the study. When the independent variables were not dichotomous, the authors would meet and discuss how to best dichotomize the data. These independent variables included age (<25 years, ≥25 years). Age was dichotomized in accordance with the definition of a nontraditional student according to the National Center for Education Statistics [10]. Other independent variables included gender (female, male), Spanish speaking (yes, no), AZ Resident (yes, no), attended AZ high school (yes, no), pharmacist in the family (yes, no), first generation college applicant (yes, no), previous applicant to the college (yes, no), Pharmacy College Admissions Test (PCAT) biology score (70–100, 0–69), PCAT chemistry score (70–100, 0–69), PCAT composite score (70–100, 0–69), PCAT math score (70–100, 0–69), PCAT reading score (70–100, 0–69), science GPA (3.5–4.0, 3.0–3.49, <3), and non-science GPA (3.5–4.0, 3.0–3.49, <3). A PCAT score of 70 was chosen as a cutoff for the PCAT variables since this is considered a competitive PCAT score [11]. Elements of the applicant’s letters of recommendation were scored by the admission’s office during the applicant’s admission cycle. These elements were tested as independent variables, including the letter writer’s assessment of the applicant’s adaptability (3–5, 0–2.9), empathy (3–5, 0–2.9), their ability to think ahead (3–5, 0–2.9), honesty (3–5, 0–2.9), leadership (3–5, 0–2.9), if the applicant was liked by their colleagues (3–5, 0–2.9), maturity (3–5, 0–2.9), the applicant’s autonomy (3–5, 0–2.9), and if the recommender would suggest the applicant for admission (3–5, 0–2.9). The dependent variable was admission status (admitted to the COP versus not admitted to the COP).

Differences in the characteristics of Hispanic versus not Hispanic applicants were compared using chi-square tests or Fisher’s exact test as appropriate. An adjusted logistic regression model was constructed to identify the variables (described above) that were associated with COP admission status, where not admitted to the COP served as the reference group. The alpha level (set a priori) to determine statistical significance was 0.05. All analyses were conducted using SAS Studio (SAS Institute Inc., Cary, NC, USA). The University of Arizona Institutional Review Board approved this study (IRB #2009061709, 23 September 2020).

## 3. Results

This study included a total of 2096 applicants, of which 272 were Hispanic and 1824 were not Hispanic. Most applicants in the study had the following characteristics: aged <25 years, female, not Spanish speaking, AZ resident, attended AZ high school, did not have a pharmacist in the family, were not first-generation college applicants, had not applied to the COP before, had PCAT scores ≥ 70, recommender’s scores ≥ 3, and science and non-science GPAs ≥ 3.5. There were significant differences between individuals who were Hispanic and those who were not Hispanic (*p* < 0.05) for several variables (Table 1, Table 2 and Table 3).

Hispanic ethnicity (Hispanic versus non-Hispanic) was not associated with admission status (adjusted odds ratio (AOR) = 1.202, 95% confidence interval (CI) = 0.841, 1.719). Characteristics significantly associated with admission to pharmacy school were: age < 25 versus ≥ 25 years (AOR = 2.273, 95% CI = 1.783, 2.907); female versus male gender (AOR = 1.536, 95% CI = 1.224, 1.927); attended high school within versus outside Arizona (AOR = 1.743, 95% CI = 1.289, 2.359); previous application to the college versus no previous application (AOR = 0.675, 95% CI = 0.522, 0.871); PCAT biology score ≥ 70% versus < 70% (AOR = 1.696, 95% CI = 1.321, 2.177); and PCAT chemistry score ≥ 70% versus < 70% (AOR = 1.618, 95% CI = 1.258, 2.080). The logistic regression model had a c-statistic of 0.720 and Wald statistic of <0.0001 (Table 4, Table 5 and Table 6).

## 4. Discussion

This study was completed to determine if the admissions criteria used at the COP could be associated with the disparity observed between the number of Hispanic people in Arizona and the number of Hispanic students enrolled into the COP. This study found that ethnicity was not associated with pharmacy school admissions at the COP. However, being 25 years or younger, female, attending an Arizona high school, having a previous application to the college, and having higher PCAT biology and chemistry scores were associated with pharmacy school admissions. These results may reflect that the admissions process relies on quantitative metrics versus a more qualitative review of the applicant. Utilizing quantitative measures to predict proficient health care professionals is common practice in the healthcare fields. To assess pharmacy graduate competency, students are required to pass the North American Pharmacist Licensure Examination (NAPLEX). Studies have found that performance on the PCAT, pharmacy GPA, and pre-NAPLEX scores are predictors of student performance on the NAPLEX [12,13,14,15]. However, there has also been research that has shown that the conventional social context of testing adversely affects minority students’ performance on these measures [16,17,18]. Recently, there has been a shift in utilizing PCAT scores for admissions [19]. According to PharmCAS, the PCAT is either optional or not required in more than 80% of colleges [20]. PCAT scores were required at the COP during the study period; however, the COVID-19 pandemic has impacted the availability of PCAT testing in 2020 and 2021 and the COP does not currently require or consider the PCAT score for admissions. This change may further diversify the student body by encouraging the admissions criteria to become based on qualitative measures; however, it is just one of the many quantitative measures being used to evaluate applicants. 

Davidson and Lewis showed that URM students admitted to medical school programs under affirmative action programs are as likely as their peers to graduate from medical school, pass licensing boards, and enter practices, regardless of having lower quantitative measures, such as MCAT scores and college GPAs [21]. The study includes ethnicity as a criterion for admissions to compensate for past societal discrimination. Using ethnicity as a criterion may diversify the profession, lead to practitioners that are more likely to practice in areas that are medically underserved and have higher percentages of minority patients [21]. Additionally, this study highlights the need to establish programs that encourage URM students to apply for pharmacy school. There were only 272 applicants that were Hispanic out of 2096 total applicants (13%). Historically, schools of pharmacy tend to recruit applicants from the undergraduate level [22]. Since the University of Arizona’s undergraduate racial/ethnic profile also differs from the state population, this method of recruitment would require recruitment efforts or programs to be targeted towards students that are Hispanic. Being an Arizona high school graduate led to greater odds in admissions. As discussed by McLaughlin and colleagues, creating opportunities for pre-college students to be exposed to the healthcare professions and creating pipeline programs targeting students identifying as racial and ethnic minorities will increase the number of URM students interested in pursuing a future career in pharmacy [23]. Recent efforts include outreach to high school students, particularly those in predominantly Hispanic areas, increasing the likelihood of admitting a more diverse population of students.

Developing qualitative measures to evaluate applications and establishing programs to recruit pre-college Arizona students that are Hispanic will ensure future COP cohorts will better reflect and serve the diversity of the region and the university’s designation as an HSI.

Excluding incomplete applications may be a limitation to this study. Gaining understanding as to the characteristics associated with students that start applications and do not complete them may provide additional insight into the resources available to students that are interested in applying to the COP. For example, the findings may suggest that more students from URM populations may start the application but are unable to complete it in its entirety. Given that this study used data from one college of pharmacy, the findings may not be generalizable to other colleges of pharmacy.

Lastly, it is important to note that admissions are one aspect of understanding the lack of diversity, as it relates to students that are Hispanic, at the COP. Further studies are required to gain additional insight into this disparity.

## 5. Conclusions

This study found that at this College of Pharmacy, ethnicity was not associated with pharmacy school admission and identified several characteristics associated with pharmacy school admission status. This study highlights the need for intervention prior to the pharmacy school application process and in the admissions process to ensure future cohorts better reflect the diversity of the region.

## Figures and Tables

**Table 1:** Characteristics of study subjects stratified by Hispanic status.

Variable	Hispanic (N = 272) N (%)	Not Hispanic (N = 1824) N (%)	*p*
Age, years			0.0394
<25	158 (58.1)	1177 (64.5)	
≥25	114 (41.9)	647 (35.5)	
Gender			0.6774
Female	166 (61.0)	1089 (59.7)	
Male	106 (39.0)	735 (40.3)	
Spanish Speaking			<0.0001
Yes	128 (47.1)	190 (10.4)	
No	144 (52.9)	1634 (89.6)	
AZ Resident			0.0218
Yes	241 (88.6)	1516 (83.1)	
No	31 (11.4)	308 (16.9)	
Attended AZ high school			<0.0001
Yes	220 (80.9)	1222 (67.0)	
No	52 (19.1)	602 (33.0)	
Pharmacist in the family			0.1348
Yes	36 (13.2)	307 (16.8)	
No	236 (86.8)	1517 (83.2)	
First generation college applicant			<0.0001
Yes	125 (46.0)	442 (24.2)	
No	147 (54.0)	1382 (75.8)	
Applied to the COP before			0.8577
Yes	56 (20.6)	367 (20.1)	
No	216 (79.4)	1457 (79.9)	

Differences between groups compared with Chi-square test or Fisher’s exact test (as appropriate). COP = College of Pharmacy; GPA = grade point average; PCAT = Pharmacy College Admissions Test.

**Table 2:** PCAT and GPA scores of study subjects stratified by Hispanic status.

Variable	Hispanic (N = 272) N (%)	Not Hispanic (N = 1824) N (%)	*p*
PCAT biology score			0.0046
70–100	136 (50.0)	1078 (59.1)	
0–69	136 (50.0)	746 (40.9)	
PCAT chemistry score			<0.0001
70–100	136 (50.0)	1262 (69.2)	
0–69	136 (50.0)	562 (30.8)	
PCAT composite score			<0.0001
70–100	109 (40.1)	163 (59.9)	
0–69	1061 (58.2)	763 (41.8)	
PCAT math score			<0.0001
70–100	71 (26.1)	858 (47.0)	
0–69	201 (73.9)	966 (53.0)	
PCAT reading score			0.0021
70–100	75 (27.6)	678 (37.2)	
0–69	197 (72.4)	1146 (62.8)	
Science GPA			0.0071
3.5–4.0	140 (51.5)	1121 (61.5)	
3.0–3.49	128 (47.1)	684 (37.5)	
<3	4 (1.5)	19 (1)	
Non-science GPA			0.0006
3.5–4.0	184 (67.7)	1413 (77.5)	
3.0–3.49	82 (30.2)	396 (21.7)	
<3	6 (2.2)	15 (0.8)	

Differences between groups compared with Chi-Square test or Fisher’s exact test (as appropriate). COP = College of Pharmacy; GPA = grade point average; PCAT = Pharmacy College Admissions Test.

**Table 3:** Interview ratings of study subjects stratified by Hispanic status.

Variable	Hispanic (N = 272) N (%)	Not Hispanic (N = 1824) N (%)	*p*
Adaptability			0.5665
3–5	270 (99.3)	1799 (98.6)	
0–2.9	2 (0.7)	25 (1.4)	
Ability to think ahead			0.3304
3–5	264 (97.1)	1746 (95.7)	
0–2.9	8 (2.9)	78 (4.3)	
Empathy			0.2761
3–5	269 (98.9)	1786 (97.9)	
0–2.9	3 (1.1)	38 (2.1)	
Honesty			0.1286
3–5	270 (99.3)	1821 (99.8)	
0–2.9	2 (0.7)	3 (0.2)	
Leadership			0.6316
3–5	264 (97.1)	1760 (96.5)	
0–2.9	8 (2.9)	64 (3.5)	
Likeability			0.1296
3–5	270 (99.3)	1786 (97.9)	
0–2.9	2 (0.7)	38 (2.1)	
Maturity			0.2787
3–5	270 (99.3)	1818 (99.7)	
0–2.9	2 (0.7)	6 (0.3)	
Suggest applicant for admission			0.0667
3–5	255 (93.8)	1647 (90.3)	
0–2.9	17 (6.3)	177 (9.7)	
Autonomy			0.3843
3–5	272 (100)	1818 (99.3)	
0–2.9	0 (0)	12 (0.7)	

Differences between groups compared with Chi-Square test or Fisher’s exact test (as appropriate). COP = College of Pharmacy; GPA = grade point average; PCAT = Pharmacy College Admissions Test.

**Table 4:** Association of variables on admitted versus not admitted status among University of Arizona pharmacy school applicants.

Variable	Adjusted Odds Ratio (95% CI)
Ethnicity	
Hispanic	1.202 (0.841, 1.719)
Non-Hispanic	Reference
**Age, years**	
**<25**	**2.273 (1.783, 2.907)**
**≥25**	**Reference**
**Gender**	
**Female**	**1.536 (1.224, 1.927)**
**Male**	**Reference**
Spanish Speaking	
Yes	1.306 (0.938, 1.819)
No	Reference
AZ Resident	
Yes	0.759 (0.521, 1.106)
No	Reference
**Attended AZ high school**	
**Yes**	**1.743 (1.289, 2.359)**
**No**	**Reference**
Pharmacist in the family	
Yes	0.948 (0.707, 1.271)
No	Reference
First generation college applicant	
Yes	1.134 (0.884, 1.455)
No	Reference
**Applied to the COP before**	
**Yes**	**0.675 (0.522, 0.871)**
**No**	**Reference**

CI = confidence interval; COP = College of Pharmacy; GPA = grade point average; PCAT = Pharmacy College Admissions Test. **Bold** indicates the variable was significantly associated with admission to the college of pharmacy.

**Table 5:** Association of PCAT and GPA variables on admitted versus not admitted status among University of Arizona pharmacy school applicants.

Variable	Adjusted Odds Ratio (95% CI)
**PCAT Biology Score**	
**70–100**	**1.696 (1.321, 2.177)**
**0–69**	**Reference**
**PCAT Chemistry Score**	
**70–100**	**1.618 (1.258, 2.080)**
**0–69**	**Reference**
PCAT Composite Score	
70–100	1.214 (0.888, 1.659)
0–69	Reference
PCAT Math Score	
70–100	1.199 (0.934, 1.539)
0–69	Reference
PCAT Reading Score	
70–100	0.895 (0.684, 1.172)
0–69	Reference
Science GPA	
3.5–4.0	0.901 (0.326, 2.494)
3.0–3.49	0.680 (0.248, 1.864)
<3	Reference
Non-science GPA	
3.5–4.0	1.961 (0.776, 4.952)
3.0–3.49	1.612 (0.635, 4.094)
<3	Reference

CI = confidence interval; COP = College of Pharmacy; GPA = grade point average; PCAT = Pharmacy College Admissions Test. **Bold** indicates the variable was significantly associated with admission to the college of pharmacy.

**Table 6:** Association of interview rating scale variables on admitted versus not admitted status among University of Arizona pharmacy school applicants.

Variable	Adjusted Odds Ratio (95% CI)
Adaptability	
3–5	1.595 (0.583, 4.365)
0–2.9	Reference
Ability to think ahead	
3–5	0.929 (0.498, 1.734)
0–2.9	Reference
Empathy	
3–5	1.136 (0.489, 2.638)
0–2.9	Reference
Honesty	
3–5	1.817 (0.246, 13.402)
0–2.9	Reference
Leadership	
3–5	1.288 (0.692, 2.398)
0–2.9	Reference
Likeability	
3–5	1.040 (0.451, 2.400)
0–2.9	Reference
Maturity	
3–5	0.240 (0.021, 2.726)
0–2.9	Reference
Suggest applicant for admission	
3–5	1.181 (0.786, 1.775)
0–2.9	Reference
Autonomy	
3–5	0.887 (0.201, 3.913)
0–2.9	Reference

CI = confidence interval; COP = College of Pharmacy; GPA = grade point average; PCAT = Pharmacy College Admissions Test. **Bold** indicates the variable was significantly associated with admission to the college of pharmacy.

## Data Availability

Data are available from the corresponding author upon reasonable request.
